# I am afraid, so I buy it! The effects of anxiety on consumer assimilation and differentiation needs amongst individuals primed with independent and interdependent self-construal

**DOI:** 10.1371/journal.pone.0256483

**Published:** 2021-09-01

**Authors:** Dariusz Drążkowski, Maciej Behnke, Lukasz D. Kaczmarek

**Affiliations:** Faculty of Psychology and Cognitive Science, Adam Mickiewicz University, Poznan, Wielkopolska, Poland; Bucharest University of Economic Studies, ROMANIA

## Abstract

Individuals tend to satisfy their assimilation needs by purchasing products that bear a specific group identity. Such products might be preferred when an individual is threatened because anxiety increases affiliative needs. In contrast, individuals might be more attracted to unique-design products when they feel less anxious. We examined the impact of anxiety on assimilation and differentiation needs amongst consumers primed with independent and interdependent self-construal. We expected that anxiety would produce stronger assimilation needs and show a weaker preference for unique products. In Study 1 (*N* = 110), we found that individuals in the anxiety-inducing condition decreased their evaluation of unique products and exhibited stronger assimilation needs. Independents who felt anxiety reacted with a reduced preference for group-linked products. Study 2 (N = 102) found that introducing an anxiety-decreasing agent (vanilla scent) after a social identity threat reduced differentiation needs and preference for unique products. Physiological data showed that the social identity threat increased sympathetic arousal, but the vanilla scent did not have a soothing effect on physiological reactivity. Overall, this work showed that both anxiety and vanilla scent reduced consumer need for differentiation. Furthermore, for independents, anxiety reduced assimilation needs. We found novel determinants of assimilation/differentiation needs with implications for advertising and retailing products with a unique design.

## Introduction

Some consumers buy an iPhone just because their friends already have one, which expresses their need for assimilation with others [1]. At the same time, consumers also buy unique smartphone covers, which none of their friends have, to express their need to differentiate from others [2]. Indeed, differentiation and assimilation needs are independent [3]. Thus, individuals satisfy both needs simultaneously through consumer behaviors [4]. One factor that influences the assimilation/differentiation need is emotions [5]. Emotions are central to the actions of consumers and marketers [6]. In particular, anxiety is proposed to influence differentiation [7] and assimilation needs [8]. Since anxiety appeals is a common marketing strategy [9, 10] it is essential to determine how anxiety influences the intensity of assimilation/differentiation needs observed in consumer behavior. Furthermore, the effects of anxiety on assimilation needs depend on consumer self-construal. When anxiety is elicited by social identity threat, individuals with independent (interdependent) self-construal demonstrate decreased (increased) assimilation [5]. Thus, in this project, we aimed to examine the effects of anxiety on evaluating products associated with assimilation (group-linked products) and differentiation needs (unique products) among consumers with different self-construal types. We aimed to integrate relationships between emotional (anxiety) and self-related (self-construal) factors influencing assimilation/differentiation processes in consumption.

### Assimilation, differentiation, and consumer behaviors

Doing what is socially approved and belonging to groups are the basic human needs [1]. Assimilation with the group allows individuals to construct their social identities [11], expressed via material possessions [12]. Consumers may also conform to other group members’ choices due to the need to belong [13]. Thus, consumers incorporate the brands consistent within-group into their self-concept [14].

Contrary, the need for uniqueness indicates consumers’ need to differentiate themselves from others and be seen as one of a kind [15]. Consumers often stand out by buying products to express their uniqueness [16] or to diverge from the outgroup [2]. Individuals with a high need for uniqueness select products that are rarely chosen by others [17] and desire to possess products that are unique [18], scarce [19], or unconventional [20].

### Anxiety and differentiation

Emotions determine whether individuals prefer to assimilate or differentiate from others via consumer choices [5]. The affect-as-information framework [21] states that individuals use current emotions as sources of information when evaluating target objects because they perceive these feelings to contain valuable judgmental information. Emotions can influence product evaluation by having their source either in the target itself (e.g., being happy after purchasing a discounted product) or in some irrelevant contextual factor (e.g., shopping while being sad after watching a movie) [22].

Anxiety is a discrete emotion that influences assimilation/differentiation needs [7, 8]. Anxious individuals display high uncertainty and strong motivation to protect themselves [23]. Thus, anxious consumers prefer products that reduce risk and uncertainty [24] and avoid products that would typically satisfy consumer’s need for differentiation [18], including controversial [7, 25], scarce [26], and extremely novel options [27]. According to the affect-as-information framework [21], feeling anxiety (as a source of information) motivates an individual to avoid uncertainty and to use risk-averse heuristics [24]. Since the process of differentiation from other people is related to taking a risk and the individual cannot be sure of social evaluation of his/her expression of the sense of uniqueness [15], it can be expected that feeling anxiety inhibits differentiation processes. Therefore the following hypothesis was proposed:

H1: Anxiety decreases consumers’ differentiation needs.

### Anxiety, self-construal, and assimilation

Anxiety motivates individuals to connect with others to cope with a threatening situation [28]. Therefore anxious consumers’ purchasing decisions are influenced by social information [26, 29]. Anxiety also motivates consumers to choose products that demonstrate affiliation [1, 30]. However, some individuals do not increase their assimilation needs when facing a threat because the impact of anxiety on assimilation/differentiation needs depends on consumers’ self-construal [5].

Individuals can be divided into two groups according to their dominant type of self-construal. Individuals with independent self-construal (independents) see themselves as autonomous, distinct from the group, and unique [31]. Individuals with interdependent self-construal (interdependents) see themselves as connected to others and define their self in terms of social roles, relationships, and group memberships. When social identity is threatened, independents demonstrate decreased assimilation, which manifests as lower ratings of group-linked products [5]. In contrast, threatened interdependents demonstrate increased assimilation, which manifests as higher ratings of group-linked products [5]. Thus, independents attempt to restore positive self-worth under social identity threat by avoiding association with a threatened identity. At the same time, interdependents satisfy assimilation needs by activating their social identities, including threatened ones, to satisfy social motive to belong. A similar pattern of results was obtained by Vohs and Heatherton [32]. They showed that under personal identity threat (i.e., ego threat), independents decreased assimilation tendencies (i.e., they behaved in such a way that they were perceived as less likable). At the same time, interdependents increased assimilation (i.e., their likeability increased). Thus, whether social [5] or personal [32], identity threat is aroused, similar patterns in the responses of individuals with different self-construal emerge, i.e., under identity threat, interdependents become more interdependent with groups, and independents become more independent from groups. Since both types of threat lead to increased anxiety [33–35], we expect that anxiety may affect changes in the intensity of assimilation processes. From the perspective of affect-as-information theory, the self-construal may serve to direct the information resulting from experienced anxiety. Individuals feeling anxious are motivated to seek safety. The research results on various forms of identity threat discussed above suggest that interdependent individuals experiencing anxiety might seek safety within the affiliation group, whereas independent individuals experiencing anxiety might look for security outside the affiliation group.

We propose the following effect of anxiety on the assimilation processes:

H2: Anxiety increases consumers’ assimilation needs for interdependents (H2a), and decreases consumers’ assimilation needs for independents (H2b).

We do not expect that the self-construal moderates the intensity of the differentiation under anxiety situation, even despite that independent’s goals are to differentiate from others [36] and that they prefer more unique products than interdependents [37]. We propose that both, independent and interdependent, anxious consumers reduce their differentiation need as they are primarily motivated to reduce risk [24, 25]. We did not find empirical ground to suggest an increase in differentiation of independents in an anxiety situation. The assumption that some individuals (independents) can simultaneously tend to decrease differentiation and assimilation needs in an anxiety-provoking situation might be regarded counterintuitive because these two needs are conflicting [3]. However, previous studies found that individuals can simultaneously satisfy these conflicting needs in a single behavior. For instance, consumers can assimilate to their group by choosing a brand symbolizing group membership while differentiating by selecting a unique color of a product [4]. Since one behavior can satisfy high levels of both the assimilation and differentiation needs, the intensity of both needs can change simultaneously in the same direction.

### The present research

We conducted two multi-method studies to examine the influence of anxiety on assimilation/differentiation needs differs among consumers with different self-construal types. In Study 1 & 2, we manipulated independent/interdependent self-construal with priming. In line with previous studies (e.g. 4, 5) we accounted for the assimilation needs with the intention to purchase and preference for groups-linked products and the need to belong. We accounted for differentiation needs with measures of intention to purchase and preference for unique products and need for uniqueness. We elicited anxiety with film clips (Study 1) and social threat procedures (Study 2). We measured self-reported and physiological responses to anxiety. We accounted for physiological measures because anxiety activates the sympathetic nervous system, mobilizing resources necessary for action [38]. This addresses recommendations to integrate psychophysiology with consumers’ experience and behavior [39]. Finally, in Study 2, we aimed to elicit anxiety and reduce it in some participants using soothing olfactory cues (vanilla scent) used in previous consumer research [10].

## Study 1

The aim of Study 1 was to examine whether consumers’ self-construal moderates the effects of anxiety on the assimilation/differentiation needs. Thus, the study had a 2 (anxiety vs. neutral condition) × 2 (independent vs. interdependent self-construal) between-subjects experimental design. We manipulated self-construal via a priming technique. We elicited anxiety with film clips and monitored the effectiveness of the manipulation with self-reports and physiological measures. We run two pilot studies to support the validity of the priming technique and stimuli related to assimilation/differentiation needs.

## Materials and methods

### Participants

The participants were 110 undergraduates (65 women) aged between 18 and 26 years old (*M* = 20.35, *SD* = 1.74). Volunteers were admitted into the study on condition that they did not meet any exclusion criteria: significant health problems, use of medications, prior diagnosis of cardiovascular disease or hypertension. Participants were instructed to avoid eating for at least one hour before the experiment and to refrain from physical exercise and intake of caffeine, nicotine, alcohol, or non-prescription drugs for at least two hours before the experiment. Each participant received a cinema ticket. The Institutional Ethics Committee at Faculty of Psychology and Cognitive Science, Adam Mickiewicz University approved the study. Data used in this study are available from the Open Science Framework (https://osf.io/5kvup/?view_only=2fd63096f87248b4890db80ce87d33d9).

#### Procedure

Participants were tested individually in a sound-attenuated and air-conditioned room. Upon arrival in the lab, participants provided written informed consent and were told that they would be participating in two unrelated studies. The first study’s purpose was presented as determining the relationship between language orientation and the psychophysiological reactions to film clips. The purpose of the second study was presented as evaluating consumer products. Participants were randomly assigned to one of four conditions: anxiety induction-independence priming (*n* = 28); anxiety induction-interdependence priming (*n* = 28); control-independence priming (*n* = 26); control-interdependence priming (*n* = 26). The biosensors were attached, and the experiment began with a five-minute resting. The experiment was run in the e-Prime 2.0 Professional Edition environment (Psychology Software Tools).

After baseline, participants’ university identity had been made salient by asking them to respond to questions from a Collective Self-Esteem Scale [40]. Next, self-construal was manipulated via the Scrambled Sentences Test. Participants created grammatically correct and meaningful sentences using four words out of a set of five words presented in a random order [41]. There was one correct sentence for each set of five words. After creating the sentence in their mind, participants typed the unused word. The mentally constructed sentences and the unused words were chosen to prime a particular self-construal. For example, "very, am, isolation, assertive, I" primed the independent self-description: ’I am very assertive’. There were 17 sentences that activated independent self or 17 sentences that activated interdependent self. The test also included three neutral sentences. The results of a preparation study indicated that this test activated the independent and interdependent self-construal (S1 File).

After priming independent or interdependent self-construal, we elicited anxiety or presented a neutral material via validated film clips [42, 43]. We elicited anxiety with excerpts from movies’ The Blair Witch Project’ [42] and ’A Tale of Two Sisters’ [43]. For the neutral condition, we used clips from ’The Lover’ and ’Three Colours: Blue’ [42]. Each sequence lasted 3:41 minutes. After the anxiety manipulation, participants reported a current level of social needs.

Finally, participants evaluated six pairs of products. Each pair included one product awarded for its innovative design and one product branded with the participants’ university logo symbolizing group membership. We asked participants to evaluate each product for their preference and purchasing intention. Photos of the products in each pair were presented simultaneously in a counterbalanced order. The instructions indicated that both products within each pair were matched for price and were intended for both women and men. We preselected photos of products in a preparatory study (S1 File).

At the end of the experiment, participants retrospectively evaluated the anxiety manipulation effectiveness, answered a probe question to determine whether they had guessed the study’s true purpose (none had), and were debriefed.

### Measures

#### Assimilation/differentiation needs

We assessed assimilation/differentiation needs by measuring preference for products and purchase intentions. We used three 9-point bipolar scales to assess preference for products. The scales’ left and right ends were labeled “*unfavorable”* vs. “*favorable”*, “*dislike”* vs. “*like”*, and “*bad”* vs. “*good”* [5]. We calculated the difference scores obtained by subtracting the preferences for the neutral product (α = .84) from the preference for the unique (α = .85) or group-linked product (α = .89) within each pair [5]. Assimilation was represented by a higher index of relative preference for university-linked products. In contrast, differentiation was represented by a lower index of relative preference.

Participants also expressed their intention to purchase products by distributing ten points between products from each of the six pairs. For example, 3 points to one product in a pair implied 7 points allocated to another. With this method, we accounted for a relative purchase intention to purchase unique (α = .64) and group-linked products (α < .50; due to low Cronbach’s alpha, we did not analyze this variable). Differentiation was represented by a stronger intention to buy unique products.

#### Social needs

We measured the need for uniqueness with four items [44], e.g., "*I prefer being ____ different from other people*" (participants insert one of the five presented adjectives; α = .87), the need to belong with three items scale [5], e.g., "*I want other people to accept me*" (α = .60), and the need for self-worth with five items scale [5], e.g., "*I feel I am not doing well*" (α = .87). The scales ranged from 1 = (*not at all*) to 7 = (*extremely*).

#### Emotions

For the manipulation check, we measured self-reported emotions and physiological responses. First, we checked the anxiety manipulation effectiveness by asking participants to indicate retrospectively the extent to which they had felt “*threatened”*, “*concerned”*, “*calm”*, “*nervous”*, “*upset”*, “*frightened”*, “*jittery”*, and “*uncertain”* while watching the movie clips. The scales range from 1 = (*not at all*) to 5 = (*extremely*) (α = .92). We used the scale as the measure of threat.

Second, we tested whether induction of anxiety was successful using cardiovascular activity and skin conductance. We measured cardiovascular activity using electrocardiogram (ADInstruments, New Zealand) and hemodynamic responses (Finapres Medical Systems, Netherlands). Electrocardiogram was recorded at 1000 Hz with BioAmp and Powerlab 16/35 AD converter (ADInstruments, New Zealand). We used Ag–AgCl surface electrodes placed on the chest and stored the signal on a computer with other physiological variables using data acquisition and analysis system (LabChart 8.1; ADInstruments, New Zealand). The signal was amplified and band-pass filtered at 0.1–40 Hz. We used the ECG Analysis 2.0 module in LabChart to calculate heart rate (HR) based on the ECG signal. Skipped or spurious beats were identified by flagging intervals larger than 1,200 ms or smaller than 400 ms intervals. Next, we visually inspected the ECG signal and in some cases inserted or removed R spikes as appropriate.

For hemodynamic responses, we recorded systolic blood pressure (SBP), diastolic blood pressure (DBP), cardiac output (CO), stroke volume (SV), and total peripheral resistance (TPR) continuously using Finometer MIDI (Finapres Medical Systems, Netherlands). Finger arterial pressure waveforms were recorded with finger cuffs, recorded on digits III of the left hand. The data were analyzed with BeatScope 2.0 (Finapres Medical Systems, Netherlands). The BeatScope 2.0 provides values of SBP, DBP, CO, SV, and TPR, for each heartbeat, based on the raw signal from Finometer MIDI. Finometer uses the volume-clamp method first developed by Penaz [45] to measure finger arterial pressure waveforms with finger cuffs.

We measured tonic skin conductance level (SCL) with the GSR Amp (ADInstruments, New Zealand) at 1000 Hz and reported in micro siemens (μS). We used electrodes with a contact area of 8 mm diameter filled with a TD-246 skin conductance paste attached with adhesive collars and sticky tape to the medial phalanges of digits II and IV of the left hand. Signals were zeroed for each participant before recording. We used initial baseline correction (“subject zeroing”) to eliminate the impact of the participant’s absolute level of electrodermal activity.

We used HR, SBP, DBP, CO, SV, SCL, and TPR, to measure sympathetic arousal related to emotional processing [46, 47]. We calculated mean values for two 220-s intervals: 1) last 220-s of baseline, and 2) the whole films which lasted 220 s. To operationalize physiological changes, we used reactivity scores corrected for the resting state levels; thus, we subtracted the baseline levels from the film clips. Using difference scores is a standard strategy for studying autonomic responses to psychological factors [48, 49].

### Analytic strategy

#### Manipulation check

First, we compared the level of threat elicited by film clips with a *t-*test. Second, to test whether anxiety film clips elicited greater physiological reactivity than neutral film clips, we run series of analyses of variance (ANOVA) and calculated effect sizes (*η*^*2*^).

#### Main analysis

We analyzed the assimilation/differentiation process in three steps. First, we run two-way ANOVAs 2 (Anxiety: present vs. absent) × 2 (Self-construal: independent vs. interdependent) separately for dependent variables (preference for unique products and for group-identity products; self-worth concerns, need to belong, and need for uniqueness). For significant interactions, we run simple effects analysis to test the effect of anxiety separately at two types of self-construal. Simple effects would demonstrate significant differences between anxiety and control condition for independents and interdependents. Finally, we run post-hoc comparisons using the Bonferroni correction to test differences between the control condition and the anxiety conditions separately for independents and interdependents. We also run a standard *t*-student test to report value t for a more detailed post-hoc comparison description. We ran the ANOVA analyses without assumption checks, as ANOVA is robust against the non-normality of data [50].

## Results

### Manipulation check

We found that participants who watched anxiety film clips reported higher levels of threat (*M* = 2.87, *SD* = 0.83) than participants in the control condition (*M* = 1.71, *SD* = 0.48), *t*(108) = 8.91, *p* < .001, Cohen’s *d* = 1.71. Watching anxiety-provoking film clips elicited the targeted physiological reactivity (Table 1). We observed greater increases in HR, SBP, SV, CO, and TPR in the anxiety condition than in the control condition.

**Table 1:** Physiological reactivity to film clips—Means (M), Standard Deviations (SD), results of Analysis of Variance (ANOVA).

Measure	Neutral	Anxiety	*F*	*df*	*p*	*η* ^*2*^
	*M (SD)*	*M (SD)*				
ΔHR [beats/min]	-0.94 (4.08)	1.72 (6.71)	5.77	1, 96	0.02	0.06
ΔSBP [mmHg]	4.98 (10.49)	9.51 (8.49)	5.31	1, 93	0.02	0.05
ΔDBP [mmHg]	2.61 (5.92)	3.99 (7.51)	1.00	1, 93	0.32	0.01
ΔSV [ml]	2.16 (6.97)	8.42 (6.69)	19.69	1, 92	< 0.001	0.18
ΔCO [l/min]	0.07 (0.69)	0.88 (0.96)	22.21	1, 92	< 0.001	0.19
ΔTPR [mmHg.min/l]	0.04 (0.08)	-0.05 (0.09)	21.88	1, 92	< 0.001	0.19
ΔSCL [μS]	1.18 (1.69)	1.44 (1.51)	0.64	1, 96	0.42	0.01

Note. Δ = measures reported as the difference scores relative to baseline. HR = heart rate, SBP = systolic blood pressure, DBP = diastolic blood pressure, SV = stroke volume, CO = cardiac output, TPR = total peripheral resistance, SCL = skin conductance level.

### Main analysis

Descriptive statistics for self-reports are presented in Table 2. Participants in the anxiety-inducing condition had lower need for uniqueness, *F*(1, 106) = 5.17, *p* < .05, η^2^ = .05, lower preference for unique products, *F*(1, 106) = 8.18, *p* < .01, η^2^ = .07, lower intention to purchase unique products, *F*(1, 105) = 7.00, *p* = .01, η^2^ = .06, and higher need to belong, *F*(1, 106) = 6.17, *p* < .05, η^2^ = .06, than the controls. There was no significant difference between the anxiety-inducing condition and the control condition for: need for self-worth, *F*(1, 106) = .50, *p* = .48, η^2^ = .01; preference for groups-linked products, *F*(1, 106) = .94, *p* = .33, η^2^ = .01.

**Table 2:** Descriptive statistics for self-reports in Study 1.

	Anxiety			Neutral			IND -	INTER -
Measure	IND	INTER	Overall	IND	INTER	Overall	overall	overall
	(n = 28)	(n = 28)	(n = 56)	(n = 27)	(n = 27)	(n = 54)	(n = 55)	(n = 55)
	*M (SD)*	*M (SD)*	*M (SD)*	*M (SD)*	*M (SD)*	*M (SD)*	*M (SD)*	*M (SD)*
Need for self-worth	2.81	3.42	3.11	3.01	2.84	2.93 (1.33)	2.91 (1.36)	3.13 (1.46)
	(1.18)	(1.70)	(1.48)	(1.54)	(1.10)			
Need to belong	5.11	5.07	5.09	4.64	4.47	4.56 (1.90)	4.88 (1.07)	4.78 (1.22)
	(0.97)	(1.14)	(1.05)	(1.14)	(1.25)			
Need for uniqueness	2.46	2.66	2.56	3.31	2.59	2.95 (1.08)	2.88	2.63
							(0.99)	(0.90)
	(0.70)	(0.83)	(0.76)	(1.06)	(0.99)			
Preference for unique products	-2.71	-3.25	-2.98 (1.68)	-1.33	-2.45	-1.89 (2.34)	-2.03 (2.20)	-2.86 (1.92)
	(1.83)	(1.49)		(2.34)	(2.24)			
Intention to purchase unique products	3.25	3.04	3.14 (1.17)	4.36	3.46	3.91 (1.82)	3.81 (1.57)	3.24 (1.52)
	(1.13)	(1.23)		(1.77)	(1.82)			
Preference for group-linked products	-0.49	0.67	0.09	0.26	0.33	.30	-0.12	0.51
	(1.67)	(1.08)	(1.51)	(0.73)	(0.69)	(0.70)	(1.34)	(0.92)

Independents had higher preference for unique products, *F*(1, 106) = 4.73, *p* < .05, η^2^ = .04, higher intention to purchase unique products, *F*(1, 105) = 3.76, *p* = .055, η^2^ = .04), and had lower preference for groups-linked products, *F*(1, 106) = 8.41, *p* < .01, η^2^ = .07, than interdependents. There was no significant difference between independents and interdependents for: need for self-worth, *F*(1, 106) = .67, *p* = .42, η^2^ = .01; need for uniqueness, *F*(1, 106) = 2.33, *p* = .13, η^2^ = .02; need to belong, *F*(1, 106) = .24, *p* = .63, η^2^ = .002.

**Fig 1:**
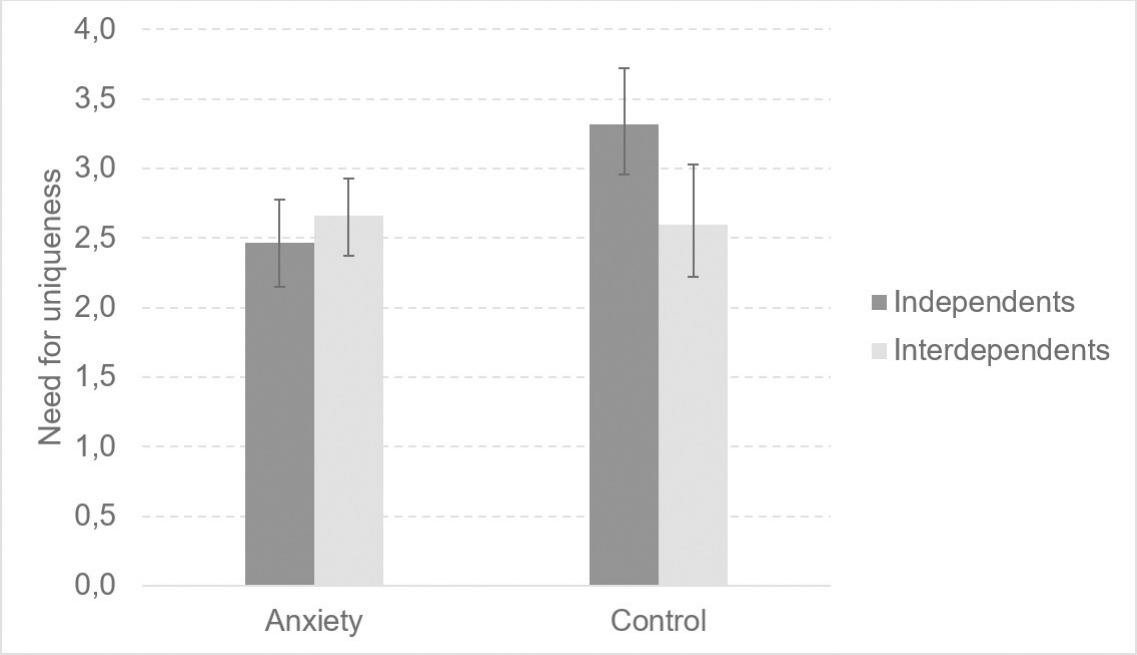
The effects of anxiety and self-construal on the preference for groups-linked products. Error bars represent standard errors estimated using the bootstrap method (95%).

**Fig 2:**
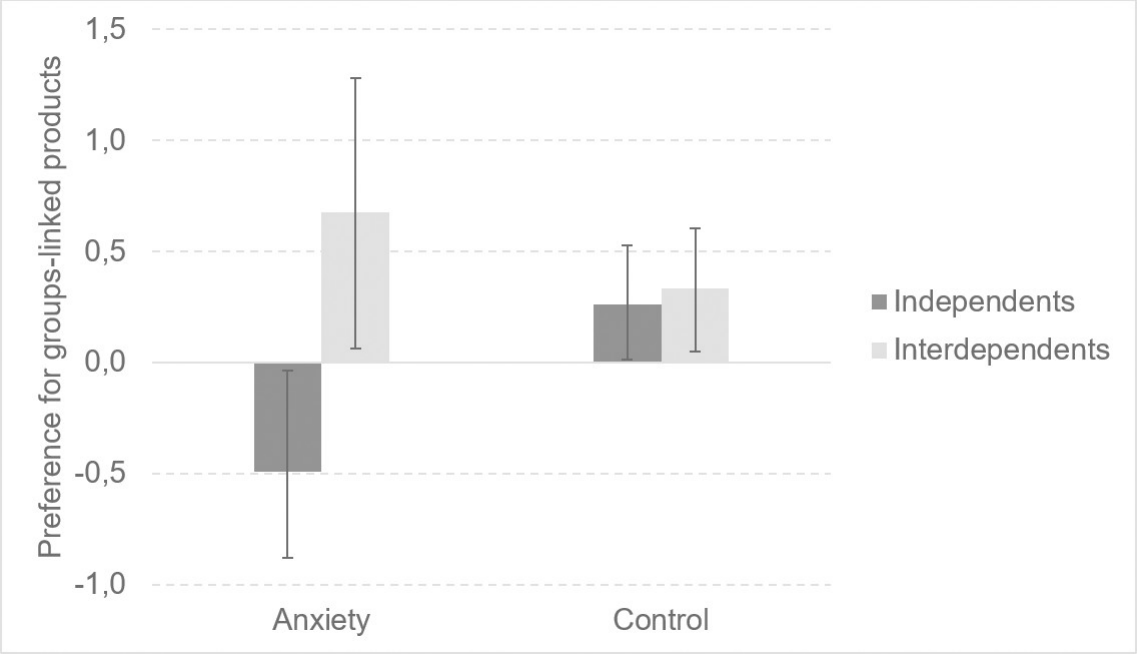
The effects of anxiety and self-construal on the need for uniqueness. Error bars represent standard errors estimated using the bootstrap method (95%).

There was an interaction of anxiety and self-construal for need for uniqueness, *F*(1, 106) = 7.12, *p* < .01, η^2^ = .06. Planned contrasts showed that independents in the anxiety-inducing condition reported lower need for uniqueness than did independents in the control condition, *t* = -3.52, *p* < .001. We also found an interaction between anxiety and self-construal for preference for groups-linked products, *F*(1, 106) = 6.61, *p* < .05, η^2^ = .06. Independents in the anxiety-inducing condition reported a lower preference for groups-linked products than did independents in the control condition, *t* = -2.19, *p* < .05. Significant interaction results are presented in Figs 1 and 2. An interaction of anxiety and self-construal did not reach statistical significance for: need for self-worth, *F*(1, 106) = 2.19, *p* = .14, η^2^ = .02; need to belong, *F*(1, 106) = 6.61, *p* < .05, η^2^ = .06; preference for unique products, *F*(1, 106) = .61, *p* = .44, η^2^ = .01; intention to purchase unique products, *F*(1, 106) = 1.41, *p* = .24, η^2^ = .01.

## Discussion

We examined how consumers’ self-construal moderate anxiety’s effects on the consumers’ assimilation/differentiation needs. We found that consumers in the anxiety-inducing condition differentiated less as manifested in a reduced preference for unique products and lower intention to purchase unique products. However, anxiety reduced the need for uniqueness only in independents. Anxiety elicitation led to increased consumers’ assimilation, as indicated by the increased need to belong. Independents reduced their assimilation after anxiety elicitation, as indexed by the preference for group-linked products. These findings partially support that self-construal moderates the assimilative reactions to anxiety [5]. These results suggest that, under anxiety, independents simultaneously feel greater need to belong, avoid products that symbolize group membership and have unique design, choosing products with a common look without symbols of membership in any group. Moreover, we replicated the effects observed in the previous studies [37] and found that consumers primed with independence preferred unique products significantly more than those primed with interdependence. With self-reports and physiological measures, we assured the effectiveness of anxiety elicitation with film clips.

## Study 2

In Study 2, we aimed to replicate and extend Study 1 by applying a different method to induce anxiety and extending emotion manipulation by using methods that reduce anxiety.

In Study 2, we induced the anxiety with the social identity threat paradigm [51, 52], which influenced assimilation/differentiation needs in previous studies [5, 53]. We applied social identity threat to induce anxiety and we used evaluations of products symbolizing threatened group membership as indicators of assimilation. This provides an opportunity to relate the study results to previous research in which the self-construal moderated changes in preference for products symbolizing threatened social identity [5]. Therefore, this study aims to extend previous research by examining whether threatened social identity without experienced anxiety leads to changes in preference for products symbolizing threatened social identity.

Study 1 showed that anxiety can change assimilation/differentiation needs in much the similar way that social identity threat does. Since anxiety mediate the link between identity threat and changes in consumer behaviors [54], reducing anxiety is likely to block consumers’ response to identity threat [52, 53]. However, no previous study tested if a reduction of anxiety after social identity threat would block changes in assimilation/differentiation needs. We expected that anxiety itself, and not the social identity threat, affects the intensification of those needs. We aimed to reduce the anxiety by exposing participants to a vanilla scent. Vanilla scent produces soothing effects that are likely to block consumers’ response to identity threat [52, 53]. Using vanilla scent as an anxiety reducer allows relating the research results to manipulation of the store atmosphere as an element of retailing [55].

We predicted the following:

H3. Reducing anxiety after exposure to a social identity threat would inhibit changes in assimilation/differentiation needs.

Thus study 2 had a 2 (vanilla vs. neutral aroma) × 2 (independent vs. interdependent self-construal) between-subjects experimental design. We expected that consumers under social identity threat in the neutral (no scent) condition would experience anxiety. In turn, they would demonstrate lower differentiation than consumers in the scented condition, where anxiety would be reduced. We also expected that interdependents (independents) in the unscented condition would demonstrate higher (lower) assimilation than in the scented condition. Finally, we expected that social identity threat would increase anxiety as indicated by self-reports and increases in physiological measures and that exposure to the vanilla scent would decrease the anxiety.

## Materials and methods

### Participants

The participants were 102 undergraduates (63 women) from Adam Mickiewicz University in Poland, aged between 18 and 26 years old (*M* = 20.27, *SD* = 1.79). We used the same exclusion criteria and preparation guidelines as in Study 1. Each participant received a cinema ticket. The Institutional Ethics Committee at Faculty of Psychology and Cognitive Science, Adam Mickiewicz University approved the study. Data used in this study are available from the Open Science Framework (https://osf.io/5kvup/?view_only=2fd63096f87248b4890db80ce87d33d9).

### Procedure

Volunteers were told that they would be participating in two unrelated studies. In the lab, participants provided written informed consent. Participants were randomly assigned to one of four conditions: vanilla scent-independent (*n* = 25); vanilla scent-interdependent (*n* = 26); control-independent independent (*n* = 26); control-interdependent (*n* = 25). Biosensors were attached, and the experiment began with a 5-minute resting period. Next, participants completed the ’Oxford Test of Professional Competence’, which was described as "*test which allows with great accuracy to predict the future level of functioning on the labour market*". They were told that the test was used in the study conducted with 19,000 students whose professional careers had been monitored. Then participants completed the same procedure as in Study 1 to prime self-construal. Next, we induced a social identity threat by providing negative group feedback based on performance on the Oxford Test of Professional Competence [56]. Participants were informed that students from their university had an average score of 13.1 out of 40, while the overall average for students from all universities was 27.3. Finally, they received a description of the characteristics associated with low scores. We supported this social identity threat procedure’s effectiveness with pilot study (S1 File). Post-manipulation physiological data (90 s) were collected while participants read the description of the characteristics associated with low scores.

Next, the experimental group received materials treated with vanilla essential oil [52, 53]. On one day, all participants received vanilla-scented questionnaires and pens (experimental condition). On the next day, all participants received unscented materials (control condition). The first page of the questionnaires assessed the social needs. Next, participants assessed the same products as in Study 1, but this time products were presented as paper prints of photographs. Participants pressed ENTER on the keyboard after completing the questionnaire; this triggered the third measurement of physiological responses (90 seconds). Finally, participants answered a probe question to determine whether they had deduced the study’s actual purpose (none had) and were debriefed.

### Measures

#### Assimilation/differentiation needs

Participants reported the same measures as in Study 1, including intention to purchase unique products (α = .70), intention to purchase group-linked products (α = .63), preference for unique products (α = .87), preference for groups-linked products (α = .87) and preference for neutral products (α = .88).

#### Social needs

Participants reported the same measures as in Study 1, including the need to belong (α = .70), need for uniqueness (α = .85) and need for self-worth (α = .87) scales.

#### Emotions

We measured the same physiological variables and used the same processing procedures as in Study 1. Physiological measures were scored using 90-s ensemble averages (last 90 seconds of resting baseline, 90 seconds after social identity threat, and 90 seconds after vanilla scent manipulation).

### Analytic strategy

#### Manipulation check

For the manipulation check, we tested whether the identity threat elicited significant physiological reactivity in both vanilla and neutral aroma conditions and whether the aroma condition influenced the recovery from the threat. Thus, we run 2 (Condition: vanilla, neutral aroma) x 3 (Time: baseline, identity threat, recovery) repeated measures ANOVA with the experimental condition as the between-subjects variables, time as the within-subjects variable, and physiological responses as dependent variables.

#### Main analysis

We used the same approach for running the primary analysis as in study 1. We run two-way ANOVAs 2 (Condition: vanilla vs. neutral aroma) × 2 (Self-construal: independent vs. interdependent) separately for dependent variables (preference for unique products and group-identity products; self-worth concerns, need to belong, and need for uniqueness). Post hoc comparisons were completed using the Bonferroni correction with an alpha level of .05

## Results

### Manipulation check

We found that the identity threat protocol elicited sympathetic arousal. Participants responded to the social identity threat with increases in SBP, DBP, SV, CO, and SCL (Table 3). Next, we observed physiological recovery in HR, CO, and TPR. Contrary to expectations, the physiological recovery was not influenced by the vanilla scent, all *p*s > .35.

**Table 3:** Differences between experimental phases—Means (M), Standard Deviations (SD), results of Repeated Measures Analysis of Variance (rm ANOVA).

Measure	Baseline	Identity Threat	Recovery Vanilla	Recovery Neutral	rm ANOVA				Post-hoc	
									Bonferroni test	
	(T1)	(T2)	(T3)	(T3)						
	*M (SD)*	*M (SD)*	*M (SD)*	*M (SD)*	*F*	*df*	*P*	*η* ^*2*^	T1-T2	T2-T3
HR [beats/min]	81.45 (11.57)	81.78 (10.94)	78.36 (10.25)	80.37	13.11	2, 172	< 0.001	0.13	^-^	***
				(9.55)						
SBP [mmHg]	115.18 (19.94)	122.43 (19.13)	119.98 (18.34)	124.4 (19.67)	20.17	2, 176	< 0.001	0.19	***	^-^
DBP [mmHg]	63.64 (11.38)	67.31 (11.36)	67.02	69.27 (13.36)	30.72	2, 176	< 0.001	0.26	***	^-^
			(9.93)							
SV [ml]	82.58 (20.16)	87.68 (19.04)	83.36 (16.95)	85.11 (18.03)	19.27	2, 172	< 0.001	0.18	***	^-^
CO [l/min]	6.59 (1.69)	7.06 (1.72)	6.47	6.78	22.20	2, 170	< 0.001	0.21	***	***
			(1.59)	(1.41)						
TPR [mmHg.min/l]	0.81 (0.18)	0.8 (0.18)	0.86 (0.2)	0.85 (0.20)	25.17	2, 170	< 0.001	0.23	^-^	***
SCL [μS]	1.44 (1.61)	2.59 (2.22)	2.34 (1.97)	3.15 (2.77)	45.86	2, 172	< 0.001	0.35	***	-

HR = heart rate, SBP = systolic blood pressure, DBP = diastolic blood pressure, SV = stroke volume, CO = cardiac output, TPR = total peripheral resistance, SCL = skin conductance level.

### Main analysis

Table 4 presents descriptive statistics for self-reports. Participants in the vanilla-scent condition had lower need for uniqueness, *F*(1, 98) = 4.44, *p* < .05, η^2^ = .04, and lower preference for unique products, *F*(1, 98) = 4.02, *p* < .05, η^2^ = .04, than the controls. There was no significant difference between the vanilla-scent condition and the control condition for: need for self-worth, *F*(1, 98) = .20, *p* = .65, η^2^ = .002; need to belong, *F*(1, 98) = .65, *p* = .42, η^2^ = .01; intention to purchase unique products, *F*(1, 98) = 2.03, *p* = .16, η^2^ = .02; preference for groups-linked products, *F*(1, 98) = 1.22, *p* = .27, η^2^ = .01; intention to purchase group-linked products, *F*(1, 98) = 1.41, *p* = .24, η^2^ = .01.

**Table 4:** Descriptive statistics for self-reports in Study 2.

	Vanilla Scent			No Scent			IND—overall	INTER—overall
Measure	IND	INTER	Overall	IND	INTER	Overall		
	(n = 25)	(n = 26)	(n = 51)	(n = 26)	(n = 25)	(n = 51)	(n = 51)	(n = 50)
	*M (SD)*	*M (SD)*	*M (SD)*	*M (SD)*	*M (SD)*	*M (SD)*	*M (SD)*	*M (SD)*
Need for self-worth	3.71 (1.52)	3.07 (1.76)	3.38 (1.51)	3.64 (1.45)	2.88 (1.48)	3.27 (1.50)	3.67 (1.47)	2.98 (1.46)
Need to belong	4.28 (1.56)	5.09 (1.26)	4.69 (1.46)	4.72 (1.57)	5.09 (1.09)	4.90 (1.35)	4.50 (1.56)	5.09 (1.17)
Need for uniqueness	2.79 (0.87)	2.48 (0.64)	2.63 (0.77)	3.18 (0.90)	2.79 (0.92)	2.99 (0.93)	2.99 (0.90)	2.63 (0.80)
Preference for unique products	-1.41 (1.88)	-2.72 (1.62)	-2.08 (1.86)	-.92 (2.81)	-1.40 (2.61)	-1.15 (2.69)	-1.16 (2.39)	-2.07 (2.24)
Intention to purchase unique products	3.77 (1.20)	3.12 (1.09)	3.44 (1.18)	4.12 (1.99)	3.61 (1.53)	3.87 (1.78)	3.95 (1.64)	3.36 (1.34)
Preference for group-linked products	0.05 (1.60)	0.77 (1.86)	0.42 (1.76)	-0.31 (1.43)	0.40 (1.77)	0.04 (1.63)	-0.14 (1.51)	0.59 (1.81)
Intention to purchase group-linked products	5.07 (1.18)	5.68 (1.28)	5.38 (1.26)	4.88 (1.13)	5.29 (1.36)	5.08 (1.25)	4.97 (1.14)	5.49 (1.32)

Independents had higher need for self-worth, *F*(1, 98) = 5.73, *p* < .05, η^2^ = .06, higher need for uniqueness, *F*(1, 98) = 4.44, *p* < .05, η^2^ = .04, higher preference for unique products, *F*(1, 98) = 3.90, *p* = .05, η^2^ = .04, and higher intention to purchase unique products, *F*(1, 98) = 3.81, *p* = .05, η^2^ = .04, than interdependents. Independents had lower need to belong, *F*(1, 98) = 4.66, *p* < .05, η^2^ = .05, preference for groups-linked products, *F*(1, 98) = 4.67, *p* = .05, *η*^2^ = .05, and intention to purchase group-linked products, *F*(1, 98) = 4.27, *p* < .05, η^2^ = .04, than interdependents.

An interaction of vanilla scent manipulation and self-construal did not reach statistical significance for: need for self-worth, *F*(1, 98) = .04, *p* = .84, η^2^ < .001; need to belong, *F*(1, 98) = .63, *p* = .43, η^2^ = .01; need for uniqueness, *F*(1, 98) = .06, *p* = .80, η^2^ = .001; preference for unique products, *F*(1, 98) = .85, *p* = .36, η^2^ = .01; intention to purchase unique products, *F*(1, 98) = .06, *p* = .81, η^2^ = .001; preference for groups-linked products, *F*(1, 98) < .01, *p* = .99, *η*^2^ < .001, and intention to purchase group-linked products, *F*(1, 98) = .15, *p* = .70, *η*^2^ = .002.

## Discussion

In Study 2, we aimed to test whether reducing anxiety by vanilla scent after exposure to a social identity threat would inhibit threat-induced changes in assimilation/differentiation. However, the vanilla scent did not effectively reduce anxiety arousal. Thus, we were unable to examine one of the aims of Study 2, i.e., showing the relationship between anxiety reduction and assimilation/differentiation needs. Despite the ineffective reduction of anxiety, consumers in the scented condition differentiated less as manifested in a reduced need for uniqueness and preference for unique products. Moreover, interdependents demonstrated stronger group assimilation (higher need to belong, preference for groups-linked products, and intention to purchase group-linked products) than independents. We also replicated some previous findings [37] by showing that independents had a higher need for uniqueness and evaluated unique products more positively than interdependents.

## General discussion

This project is the first to provide causal and multilayer evidence that anxiety influences assimilation and differentiation needs among consumers primed with different self-construal types. We focused on changes in evaluations of unique and group-linked products. To explain consumer behavior, we integrated several theoretical perspectives into one research design, including affect-as-information theory [21], self-construal theory [31], and need for uniqueness theory [15]. In Study 1, we examined how anxiety modulates consumers’ needs for assimilation/differentiation. In Study 2, we aimed to tested whether reducing anxiety by vanilla scent after exposure to a social identity threat would inhibit threat-induced changes in assimilation/differentiation needs. However, the vanilla scent did not effectively reduce anxiety arousal. Therefore we were unable to meet this objective of the study. Still, Study 2 adds new insight into identifying antecedents of differentiation need as vanilla scent reduced this need. In the pursuit of evidence on causality, we successfully activated specific aspects of self, induced anxiety, and manipulated the consumer environment’s olfactory characteristics. Advancing previous studies examining how anxiety influences consumers [25, 26], we used several physiological indicators of anxiety arousal to support emotion manipulations validity. Table 5 summarizes our findings.

**Table 5:** Summary of results of Study 1 & 2.

	Anxiety (study 1)	Vanilla Scent (study 2)
Independents	Assimilation ↓	Differentiation ↓
	Differentiation ↓	
Interdependents	Differentiation ↓	Differentiation ↓

*Note*: The above results refer only to assimilation/differentiation expressed in differences in the products evaluations. ↓ = decreased

This investigation contributed to several streams of research. Specifically, this research contributes to work on the emotional determinants of assimilation/differentiation needs among consumers. Our research extends the understanding of those needs’ emotional determinants by demonstrating that under anxiety, consumers differentiated less. In Study 1, consumers in the anxiety-inducing condition showed a lowered evaluation of unique products. According to affect-as-information theory [21] participants used anxiety as a source of information during the evaluation of unique products. Our findings provide new insight into the mechanism of differentiation by suggesting that anxious consumers may differentiate less likely because of risk-averse heuristics [24], which inhibit the evaluation of unique products. These findings also contribute to the literature on consumer uniqueness seeking [15] by identifying a new antecedent—anxiety. In line with previous studies [29], we observed that anxiety enhanced assimilation, manifesting as an increased need to belong.

Our work contributes to research on the moderating role of self-construal concerning emotion [5, 52, 57, 58]. In Study 1, self-construal moderated some reactions to anxiety. As we expected, self-construal differences occurred in assimilative reactions. In line with previous studies, anxious independents showed a reduced preference for group-linked products [5]. However, contrary to previous findings, interdependents who felt anxiety did not change their preference for group-linked products. This discrepancy suggests that in Study 1 interdependents feeling anxiety were not concerned to defend the group of belonging, which was not threatened in previous studies [5]. Therefore we did not observe changes in their evaluation of group-linked products. Under anxiety, the need to belong increased in both types of self-construal. This means that independents in the anxiety-inducing condition showed both increased need to belong and reduced preference for group-linked products. It suggests that independents tended to cope with the anxiety by seeking to connect with others, but they seemed to find it outside the group of belonging. We had not assumed that self-construal moderated differentiative reactions to anxiety. However, we found that anxiety suppressed the need for uniqueness for independents, but not for interdependents, which could be a consequence of the fact that independents are more likely to rely on feelings and hence are more impulsive consumers [59].

Study 2 contributes to the literature on olfactory effects on consumer behaviors. We found that the need for uniqueness and the preference for unique products depends on the consumer’s olfactory experience. Consumers under social identity threat exposed to the vanilla scent differentiated less. This contributes to an understanding of what kind of odors affect consumer needs [10]. Our findings suggest a mechanism explaining the vanilla effect. Namely, previous research suggested that vanilla scents affect consumer preferences via soothing effects, but they did not control for physiological arousal [52, 53]. In contrast, our results suggest that vanilla suppressed differentiation without any meaningful physiological soothing effects. Emotions are not the only mechanism by which ambient scent affects consumer behavior [60]. Scent enhances recall of product information and generates associations with a product in memory [61]. For instance, many individuals associate the scent of vanilla with home-baked cakes and holidays [55]. Thus in our study, vanilla could have activated consumers’ communal orientation orthogonal to the need for uniqueness [15]. Our research adds to the olfactory literature by suggesting that vanilla scent can affect unique products’ preferences via cognitive pathways without affecting physiological arousal. However, we emphasized that our suggestions about the mechanism of vanilla scent’s impact on the need for differentiation are limited to situations of social identity threat and to feelings of anxiety accompanying this threat.

### Practical implications

Our work demonstrates conditions under which consumers satisfy assimilation/differentiation needs. Thus, some practical implications of these effects might be considered. First, we found that anxiety discouraged consumers from purchasing unique products. It might be worthwhile to screen sales strategies of unique products to ensure they do not elicit anxiety. To illustrate, arousing anxiety caused by imaging a phone’s damage might be a counterproductive strategy of selling unique-design phone covers. It appears that in the case of products purchased for safety reasons (e.g., child safety seats), where anxiety is the leading purchase motivation [25], it is better not to design them as unique. Our findings seem to suggest that marketers might remove anxiety triggers during personal customization of products (e.g., by removing uncertainty about the technical aspects of the customization), fulfilling uniqueness needs [18]. Moreover, pro-environmental marketing might capitalize on the advantage of mixing anxiety (e.g., aroused via presentation consequences of global warming) with the common design of products instead of the unique one.

Second, we found that anxiety negatively affected the evaluation of group-linked products, but only for independents. This suggests that advertising and sales strategies of group-linked products (e.g., products with patriotic logos, clothes fashionable in peer groups) for target markets that are more independent (e.g., from highly individualistic countries) might avoid fear appeals. Our results suggest that marketers creating a group identity link may backfire under evoking consumers’ independent self-construal and arousing anxiety. Perhaps these conditions are also unfavorable for using "top seller" taglines since this strategy could be interpreted as a manifestation of assimilation [62]. We also replicated prior findings by demonstrating that independents prefer more unique products [37] and interdependents prefer more group-linked products [5]. As specialists might tailor advertising to influence consumers’ accessibility of particular self-construal [63], it would be an effective strategy to increase the fit between the type of the product and primed self-construal. For instance, to encourage the uptake of furniture with a unique design, retailers might activate consumers’ independent self-construal by using phrases such as "express your personal taste" or "furniture designed to fulfill your desires" rather than "furniture for comfortable social gatherings."

Third, we found that when the vanilla scent was present, consumers were less likely to prefer unique products over products with standard designs. However, great caution should be employed in interpreting these results as the vanilla scent was present under conditions of social identity threat, which represents unusual shopping situations. Despite these limitations our results suggest that the vanilla scent does not affect unique products’ preferences through soothing effects but rather through cognitive pathways. This suggests that stores should ensure that the cognitive associations of particular scent are congruent with the associations of the products being sold. For instance, vanilla scent, which is associated with traditional values [55], is seemingly better suited to selling classic furniture than selling the latest electronics.

### Limitations and future directions

There are limitations to these studies. First, we used a student sample. Thus our findings may not be generalizable to less educated age peers or other age cohorts. Second, our experiments were limited by using a priming technique to investigate the effects of dominating self-construal. Third, in Study 2, we were interested in the soothing effects of vanilla, and so we did not include a control condition with no exposure to social identity threat. Therefore, it is impossible to ascertain that the social identity threat procedure induced anxiety and increased physiological arousal. Fourth, the method of eliciting anxiety via the threat of a particular social identity used in Study 2, may limit the conclusions of Study 2 for the evaluation of affiliation group products to only the processes of responding to the threat of a particular social identity, and not for anxiety responses in general. Therefore, the results of Study 2 can be only referred the social identity threat situation. Fifth, in the context of social identity threat, positively assessing a product symbolizing threatened identity may pose another threat to identity at the social level, beyond the boundary of the threatened group. Sixth, some effects that we found in this project might be difficult to interpret. For instance, low uniqueness needs and low preference for group-linked products may be interpreted as contradictory. Further studies might aim to disentangle these effects introducing different research designs or measurement methods.

Our research suggests several directions for future studies, which can avoid this study’s limitations and point to new areas of research on assimilation/differentiation. First, future research could further explore the role of anxiety in assimilation/differentiation. We found that anxiety increases the need to belong and does not affect the evaluation of group-linked products, which suggests that assimilation was activated. However, it was not reflected by evaluating those products. Thus, future studies might examine if under anxiety assimilation could be manifested in preference of products different than group-linked products, as products favored by a peer [30]. Perhaps under anxiety, the need for belonging is better satisfies via a product that other consumers use. Moreover, future studies may examine the risk-averse heuristic [24] as one of the mediators between the anxiety-differentiation link.

Second, future research might consider how other emotional states, positive (e.g., gratitude) and negative (e.g., sadness) emotions, affect assimilation/differentiation among consumers with different types of primed self-construal. Mainly, since group-based emotions (anger towards outgroup) increase group identification [64], they can also affect assimilation expressed in consumer behavior.

Third, we found limited evidence for the understanding of how vanilla scents inhibit differentiation. Future studies may use other scents that reduce anxiety (e.g., lavender scent, [65]) and compare their effects with vanilla scents. Study 2 is the first, which suggest that vanilla scent impacts consumers without soothing physiological effects. Thus, future studies may consider what cognitive associations are related to a particular scent and whether these cognitive pathways explain the relationship between scent and differentiation. It would also be of interest to examine potential moderators of vanilla scent effect on differentiation, e.g., scent intensity and awareness, past experience, or scent preference [66].

## Conclusions

This project’s strength was its focus on causal links (eliciting anxiety, priming self-construals) in explaining consumers’ preferences and intentions. Moreover, we adopted a multilayer approach towards the measurement of emotions and found that physiological measures produced different results than self-reports; evidence for their more extensive adoption in consumer research. Our findings’ complexity indicates that systematic studies on consumers’ emotions are likely to reveal how psychological influences determine the choice of specific products.

## Supporting information

S1 File Pilot studies. Development of the self-construal manipulation, product selection, and social identity threat manipulation.
(DOCX)

## References

[1] Baumeister R, Leary M. The need to belong: desire for interpersonal attachments as a fundamental human motivation. PSYCHOL BULL. 1995;117(3): 497–529. 7777651

[2] Berger J, Heath C. Where consumers diverge from others: Identity signaling and product domains. J CONSUM RES. 2007;34(2): 121–134.

[3] Brewer W. The social self: On being the same and different at the same time. PERS SOC PSYCHOL B. 1991;17(5): 475–482.

[4] Chan C, Berger J, Van Boven L. Identifiable but not identical: Combining social identity and uniqueness motives in choice. J CONSUM RES, 2012;39(3): 561–573.

[5] White K, Argo JJ, Sengupta J. Dissociative versus associative responses to social identity threat: The role of consumer self-construal. J CONSUM RES. 2012;39(4): 704–719.

[6] Bagozzi RP, Gopinath M, Nyer PU. The role of emotions in marketing. J ACAD MARKET SCI. 1999;27(2): 184–206.

[7] Kugler T, Connolly T, Ordóñez LD. Emotion, decision, and risk: Betting on gambles versus betting on people. J BEHAV DECIS MAKING. 2012;25(2): 123–134.

[8] Taylor SE. Tend and befriend: Biobehavioral bases of affiliation under stress. CURR DIR PSYCHOL SCI. 2006;15(6): 273–277.

[9] Hastings G, Stead M, Webb J. Fear appeals in social marketing: Strategic and ethical reasons for concern. PSYCHOL MARKET, 2004;21(11): 961–986.

[10] Roschk H, Loureiro SMC, Breitsohl J. Calibrating 30 years of experimental research: a meta-analysis of the atmospheric effects of music, scent, and color. J RETAILING, 2017;93(2): 228–240.

[11] Tajfel H, Turner JC. An integrative theory of intergroup conflict. In: Austin WG, Worchel S, editors. The Social Psychology of Intergroup Relations. Monterey: CA: Brooks/Cole; 1979. pp. 33–48.

[12] Tinson JS, Nuttall PJ. Social collective decision making among adolescents: a review and a revamp. PSYCHOL MARKET, 2014; 31(10): 871–885.

[13] Englis BG, Solomon MR. To be and not to be: Lifestyle imagery, reference groups, and the clustering of America. J ADVERTISING. 1995;24(1): 13–28.

[14] Escalas JE, Bettman JR. Self-construal, reference groups, and brand meaning. J CONSUM RES. 2005;32(3): 378–389.

[15] Snyder CR, Fromkin HL. Abnormality as a positive characteristic: The development and validation of a scale measuring need for uniqueness. J ABNORM PSYCHOL. 1977;86(5): 518–527.

[16] Cheema A, Kaikati AM. The effect of need for uniqueness on word of mouth. J MARKETING RES. 2010;47(3): 553–563.

[17] Ruvio A. Unique like everybody else? The dual role of consumers’ need for uniqueness. PSYCHOL MARKET. 2008;25(5): 444–464.

[18] Tian KT, Bearden WO, Hunter GL. Consumers’ need for uniqueness: Scale development and validation. J CONSUM RES. 2001;28(1): 50–66.

[19] Lynn M, Harris J. Individual differences in the pursuit of self-uniqueness through consumption. J APPL SOC PSYCHOL. 1997;27(21): 1861–1883.

[20] Bloch PH. Seeking the ideal form: Product design and consumer response. J MARKETING. 1995;59(3): 16–29.

[21] Schwarz N, Clore GL. Feelings and phenomenal experiences. In: Higgins ET, Kruglanski A, editors. Social psychology: Handbook of basic principles. New York: The Guilford Press; 1996. pp. 433–465.

[22] Pham MT, Cohen JB, Pracejus JW, Hughes GD. Affect monitoring and the primacy of feelings in judgment. J CONSUM RES. 2001;28(2): 167–188.

[23] Frijda NH. Aesthetic emotions and reality. AM PSYCHOL, 1989;44(12): 1546–1547.

[24] Pham H. Continuous-time stochastic control and optimization with financial applications (Vol. 61). Berlin: Springer Science & Business Media, 2009.

[25] Raghunathan R, Pham MT. All negative moods are not equal: Motivational influences of anxiety and sadness on decision making. ORGAN BEHAV HUM DEC. 1999;79(1): 56–77. 10.1006/obhd.1999.2838 10388609

[26] Griskevicius V, Tybur JM, Gangestad SW, Perea EF, Shapiro JR, Kenrick DT. Aggress to impress: hostility as an evolved context-dependent strategy. J PERS SOC PSYCHOL. 2009;96(5): 980–994. doi: 10.1037/a0013907 19379031

[27] Pelegrín-Borondo J, Reinares-Lara E, Olarte-Pascual C, Garcia-Sierra M. Assessing the moderating effect of the end user in consumer behavior: the acceptance of technological implants to increase innate human capacities. FRONT PSYCHOL. 2016; https://doiorg/103389/fpsyg201600132 26941662 10.3389/fpsyg.2016.00132 PMC4761839

[28] Schachter S. The psychology of affiliation: Experimental studies of the sources of gregariousness. Minneapolis: University of Minnesota Press; 1959.

[29] Gino F, Brooks AW, Schweitzer ME. Anxiety, advice, and the ability to discern: Feeling anxious motivates individuals to seek and use advice. J PERS SOC PSYCHOL. 2012;102(3): 497–512. doi: 10.1037/a0026413 22121890

[30] Mead NL, Baumeister RF, Stillman TF, Rawn CD, Vohs KD. Social exclusion causes people to spend and consume strategically in the service of affiliation. J CONSUM RES. 2011;37(5): 902–919.

[31] Markus HR, Kitayama S. Culture and the self: Implications for cognition, emotion, and motivation. PSYCHOL REV. 1991;98(2): 224–253.

[32] Vohs KD, Heatherton TF. Self-esteem and threats to self: Implications for self-construals and interpersonal perceptions. J PERS SOC PSYCHOL. 2001;81(6): 1103–1118. doi: 10.1037//0022-3514.81.6.1103 11761311

[33] Jonas E, McGregor I, Klackl J, Agroskin D, Fritsche I, Holbrook C, et al. Threat and defense: From anxiety to approach. In: Olson JM, Zanna MP, editors. Advances in Experimental Social Psychology. San Diego: Academic Press; 2014. pp. 219–286.

[34] Bosson JK, Haymovitz EL, Pinel EC. When saying and doing diverge: The effects of stereotype threat on self-reported versus non-verbal anxiety. J EXP SOC PSYCHOL. 2004;40(2): 247–255.

[35] Hirsh JB, Kang SK. Mechanisms of identity conflict: Uncertainty, anxiety, and the behavioral inhibition system. PERS SOC PSYCHOL REV. 2016;20(3): 223–244. doi: 10.1177/1088868315589475 26048875

[36] Aaker J, Schmitt B. Culture-dependent assimilation and differentiation of the self: Preferences for consumption symbols in the United States and China. J CROSS CULT PSYCHOL. 2001;32(5): 561–576.

[37] Kim H, Markus HR. Deviance or uniqueness, harmony or conformity? A cultural analysis. J PERS SOC PSYCHOL. 1999;77(4): 785–800.

[38] Bradley MM, Lang PJ. Emotion and motivation. In: Cacioppo JT, Tassinary LG, Berntson GG, editors. Handbook of psychophysiology. Cambridge: University Press; 2007. pp. 581–607.

[39] Zambardino A, Goodfellow J. Being ‘Affective’ in Branding?. J MARKETING MANAG. 2007;23(1–2): 27–37.

[40] Luhtanen R, Crocker J. A collective self-esteem scale: Self-evaluation of one’s social identity. PERS SOC PSYCHOL B. 1992;18(3): 302–318.

[41] Kühnen U, Hannover B. Assimilation and contrast in social comparisons as a consequence of self-construal activation. EUR J SOC PSYCHOL. 2000;30(6): 799–811.

[42] Schaefer A, Nils F, Sanchez X, Philippot P. Assessing the effectiveness of a large database of emotion-eliciting films: A new tool for emotion researchers. COGNITION EMOTION. 2010;24(7): 1153–1172.

[43] Reynaud E, El Khoury-Malhame M, Rossier J, Blin O, Khalfa S. All negative moods are not equal: Motivational influences of anxiety and sadness on decision making. PLOS ONE. 2012;7(3): e32413. doi: 10.1371/journal.pone.0032413 22479326 PMC3316522

[44] Song D, Lee J. Balancing "We" and "I": Self-construal and an alternative approach to seeking uniqueness. J CONSUM BEHAV. 2013;12(6): 506–516.

[45] Penaz J. Photoelectric measurement of blood pressure, volume and flow in the finger. In: Digest of the 10th International Conference on Medical and Biological Engineering. Dresden, 1973,104.

[46] Kreibig SD. Autonomic nervous system activity in emotion: A review. BIOL PSYCHOL. 2010;84(3): 394–421. doi: 10.1016/j.biopsycho.2010.03.010 20371374

[47] Siegel EH, Sands MK, Van den Noortgate W, Condon P, Chang Y, Dy J, et al. Emotion fingerprints or emotion populations? A meta-analytic investigation of autonomic features of emotion categories. PSYCHOL BULL. 2018;144(4): 343–393. doi: 10.1037/bul0000128 29389177 PMC5876074

[48] Behnke M, Gross JJ, Kaczmarek LD. The role of emotions in esports performance. EMOTION. 2020. Advance online publication. 10.1037/emo0000903 33119343

[49] Kreibig SD, Samson AC, Gross JJ. The psychophysiology of mixed emotional states. PSYCHOPHYSIOLOGY; 2013;50(8): 799–811. doi: 10.1111/psyp.12064 23730872

[50] Blanca MJ, Alarcón R, Arnau J, Bendayan R. Non-normal data: Is ANOVA still a valid option? PSICOTHEMA; 2017;29(4): 552–557. doi: 10.7334/psicothema2016.383 29048317

[51] Branscombe NR, Ellemers N, Spears R, Doosje B. The context and content of social identity threat. In: Ellemers N, Spears R, editors. Social identity: Context, commitment, content. Oxford: Blackwell Science; 1999. pp. 35–59.

[52] Angel JW. A Unified Theory of Consumer Response to Self-Concept Threat. USA: PhD thesis University of Washington; 2012.

[53] Lee K, Kim H, Vohs KD. Stereotype threat in the marketplace: Consumer anxiety and purchase intentions. J CONSUM RES. 2011;38(2): 343–357.

[54] Angle JW, Forehand MR. It’s not us, it’s you: How threatening self-brand association leads to brand pursuit. INT J RES MARK. 2016;33(1): 183–197.

[55] Spangenberg ER, Grohmann B, Sprott DE. It’s beginning to smell (and sound) a lot like Christmas: the interactive effects of ambient scent and music in a retail setting. J BUS RES. 2005;58(11): 1583–1589.

[56] White K, Argo JJ. Social identity threat and consumer preferences. J CONSUM PSYCHOL. 2009;19(3): 313–325.

[57] Brockner J, Chen YR. The moderating roles of self-esteem and self-construal in reaction to a threat to the self: Evidence from the People’s Republic of China and the United States. J PERS SOC PSYCHOL. 1996;71(3): 603–615. doi: 10.1037//0022-3514.71.3.603 8831164

[58] Cheung RY, Park IJ. Anger suppression, interdependent self-construal, and depression among Asian American and European American college students. CULT DIVERS ETHN MIN. 2010;16(4), 517–525. 10.1037/a0020655 PMC3058745 21058815

[59] Hong J, Chang HH. "I" follow my heart and "We" rely on reasons: The impact of self-construal on reliance on feelings versus reasons in decision making. J CONSUM RES. 2015;41(6): 1392–1411.

[60] Lwin MO, Morrin M. Scenting movie theatre commercials: The impact of scent and pictures on brand evaluations and ad recall. J CONSUM BEHAV. 2012;11(3): 264–272.

[61] Krishna A. An integrative review of sensory marketing: Engaging the senses to affect perception, judgment and behavior. J CONSUM PSYCHOL. 2012;22(3), 332–351.

[62] Cialdini RB, Goldstein NJ. Social influence: Compliance and conformity. ANNU REV PSYCHOL. 2004;55, 591–621. doi: 10.1146/annurev.psych.55.090902.142015 14744228

[63] Minton EA, Cornwell TB, Kahle LR. A theoretical review of consumer priming: Prospective theory, retrospective theory, and the affective–behavioral–cognitive model. J CONSUM BEHAV. 2017;16(4): 309–321.

[64] Kessler T, Hollbach S (2005). Group-based emotions as determinants of ingroup identification. J EXP SOC PSYCHOL. 2005;41(6), 677–685.

[65] Kritsidima M, Newton T, Asimakopoulou K. The effects of lavender scent on dental patient anxiety levels: a cluster randomised-controlled trial. COMMUNITY DENT ORAL. 2010;38(1), 83–87. doi: 10.1111/j.1600-0528.2009.00511.x 19968674

[66] Teller C, Dennis C. The effect of ambient scent on consumers’ perception, emotions and behaviour: A critical review. J MARKETING MANAG. 2012;28(1–2), 14–36.

