# Supplementary material for: I am afraid, so I buy it! The effects of anxiety on consumer assimilation and differentiation needs amongst individuals primed with independent and interdependent self-construal
Source: PLoS One. 2021 Sep 1;16(9):e0256483. doi: 10.1371/journal.pone.0256483 (PMC8409673; doi:10.1371/journal.pone.0256483)
Supplement: S1 File — Development of the self-construal manipulation, product selection, and social identity threat manipulation. (DOCX) [file pone.0256483.s001.docx]

**S1 File**

**Development of the self-construal manipulation**

The purpose of the preparation study was to develop manipulations that would activate either the independent or the interdependent self-construal. A Scrambled Sentences Test - SST was constructed [1]. Participants' task in SST was to create a grammatically correct and meaningful sentence with four out of the five words presented in a randomized order. For each series of five words, only one correct sentence was possible to create. After creating the sentence in mind, participants were asked to type the remaining word that was not used to build the sentence. Both sentences created in mind, and the remaining words were priming particular self-construal. To prime independent self-construal, we have adopted the set of words from the Kühnen and Hannover method [1]. For example, "very, am, isolation, assertive, I" results in the independent self-description "I am very assertive'. We also adopted a set of words and sentences related to a collective content by the previous priming technique [2,3] and self-construal scale [4] to prime collective-interdependent self-construal. Two final versions of SST consisted of 17 sentences that activated independent self and 17 sentences that activated collective-interdependent self. Three neutral sentences were also selected. All the words, originally in Polish and in English translation, used in the test to prime the independent and interdependent self-construal are shown in Table 1.

**Table 1. Set of words used in the Scrambled Sentences Test to prime independent and interdependent self-construals.**

| **Priming independent self-construal (in Polish)** | **Priming independent self-construal (English translation)** |
| --- | --- |
| 1) bez względu; swoje; **niepodobny/a**; na innych; robię  2) wyłącznie; polegam; często; **odrębny/a**; na sobie  3) **ja**; nie; inni; mnie; obchodzą  4) wyjątkowy/a; **odłączam**; że; czuję; jestem  5) **mnie**; osobą; jestem; rywalizującą; bardzo  6) różne; czytać; lubię; książki; **życie**  7) **jednostką**; jestem; innych; od; niezależny/a  8) osobą; **osamotnienie**; asertywną; jestem; bardzo  9) innych; odróżniać się; **wolny/a**; od; lubię  10) poglądy; moje; **różny/a**; niezwykłe; są  11) niezwykle; samowystarczalność; **indywidualny/a**; jest; ważna  12) lepszy/a; **niezależność**; zdecydowanie; jestem; od innych  13) samodzielnie; pracować; przeważnie; lubię; **wyjątkowy/a**  14) bardzo; oglądać; lubię; naturę; **ładna**  15) nudnych; często; **zapomnę**; zadań; unikam  16) moje; autonomiczne; **rozdzielam**; interesy; są  17) wyróżniany/a; być; **osobno**; lubię; lub nagradzany/a  18) w ogóle; wrażliwy/a; jestem; **własny/a**; nie  19) **autonomia**; najlepszy/a; być; staram się; zawsze  20) **oddzielony/a**; od; często; ludzi; izoluję się | 1) regardless; own; **unlike**; on others; I do  2) exclusively; I rely; often; **separate**; on myself  3) **I**; do not; others; care about; me  4) unique; **I disconnect**; that; I feel; I am  5) **me**; person; I am; competing; very  6) different; read; me; like; books; **life**  7) **individual**; I am; others; from; independent  8) person; **solitary**; assertive; I am; very  9) other; different; **free**; from; I like  10) opinions; my; **different**; unusual; are  11) unusually; self-sufficient; **individual;** is; important  12) better; **independence;** definitely; I am; from; others  13) independent; work; mostly; I like; **unique**  14) very; watch; I like; nature; **pretty**  15) boring; often; **I forget**; tasks; I avoid  16) my; autonomous; **I separate**; interests; are  17) distinguished; be; **separate**; I like; or rewarded  18) in general; sensitive; I am; **own**; not  19) **autonomy**; best; be; I try; always  20) **separated**; from; often; people; I isolate myself |
| **Priming interdependent self-construal (in Polish)** | **Priming interdependent self-construal (English translation)** |
| 1) między; zgadzamy się; zawsze; **razem**; sobą  2) nasze; **towarzystwie**; podobne; poglądy; są  3) od innych; zwykle; lepsi; **posłuszeństwo**; jesteśmy  4) są; **zbiorowość**; od nas; oni; słabsi  5) **społeczne**; ze sobą; interesy; wspólne; mamy  6) podobne; czytać; lubimy; książki; **życie**  7) dużo; **połączony/a**; dla nas; znaczy; grupa  8) wspólnoty; należymy; **członkostwo**; większej; do  9) dla; możemy; **w zgodzie**; poświęcić się; grupy  10) z nimi; wypadamy; **zespolenie**; korzystnie; w porównaniu  11) sprawach; w wielu; różnimy się; od nich; **zbiór**  12) **nas**; w grupie; dobrze; współpracujemy; bardzo  13) swoje; **społeczność**; wzajemnie; szanujemy; opinie  14) bardzo; oglądać; lubimy; naturę; **ładna**  15) nudnych; często; **zapominać**; zadań; unikamy  16) ze sobą; zgadzamy się; chętnie; zawsze; **nasi**  17) za siebie; współodpowiedzialni; czujemy się; **harmonia**; często  18) dla; mamy; autorytetów; **skromność**; szacunek  19) zawsze; **scalony/a**; drużynę; wspieramy; naszą  20) **nam**; lubimy; działać; razem; często | 1) between; agree; always; **together**; each other  2) our; **company**; similar; views; are  3) from others; usually; better; **obedience**; we are  4) are; **collective**; from us; they; weaker  5) **social**; with each other; interests; common; we have  6) similar; read; we like; books; **life**  7) much; **connected**; for; us; means; group  8) community; we belong**; membership**; larger; to  9) for; we can; **in harmony**; sacrifice; group  10) with them; we; **combine**; favorably; compare  11) matters; in many; we differ; from them; **set**  12) **us**; in a group; well; we cooperate; very  13) our; **community**; mutual; we respect; opinions  14) very; watch; we like; nature; **nice**  15) boring; often; **forget**; tasks; we avoid  16) with; each other; we agree; willingly; always; **our**  17) for each other; co-responsible; we feel; **harmony**; often  18) for; we have; authority; **modesty**; respect  19) always; **integrated**; team; we support; our  20) **us**; we like; act; together; often |

Note: sets of five words were presented separately in random order. The non-matching word was written in the space provided. In the above presentation of the method, non-matching words were bolded. Sentences and neutral (buffer) words: items #14 and #15; neutral words: item #6.

Forty-seven undergraduates, after completion of one version of SST, responded to an abbreviated version of the Twenty Statements Task [5], in which they listed ten statements that defined "who you are" [6]. Responses were coded by two independent raters, who were blind to the priming condition. Self-definitions were coded into three categories: independent (individual physical qualities, traits, attitudes, or activities), collective-interdependent (membership in a social category), and relational-interdependent (role in an important relationship) self-descriptions [7]. Interrater reliability of codings was .95. The number of independent responses was significantly greater in the independent condition than in the collective-interdependent condition, t(45) = -2.84, p < .01, d = 0.83, and the number of collective-interdependent responses was significantly greater in the collective-interdependent condition than in the independent condition, t(45) = 2.68, p < .01, d = 0.78. The number of relational-interdependent responses was the same in both conditions (all ps > .05). The analysis supported that the SST was successful in the activation of independent and collective-independent self-construals.

**Product selection**

We selected products based on Tian et al. work [8]. First, we selected eight categories of products consumed in the public domain: water bottles, bicycle helmets, vacuum flasks, umbrellas, rucksacks, lamps, computer bowls headphones. Using internet resources, we pre-selected photos of ten products for each of the above categories, which won the prize for innovative design as judged by product design experts. We also selected ten other products with participants' university logos. These products represented group membership symbolically [9,10]. Second, ten raters (five female and five male students) evaluated all of these products for their masculinity and femininity, relevance to student life regarding use and expense, and uniqueness of their design. We selected products that were perceived as gender-neutral, affordable, and used by students. Of the list, we selected three products with the unique design (backpack, vacuum flasks, and headphones) and three products with the least unique design as university-linked products (mug, sweatshirt, pen). For the pair of these six products, photos of counterparts products of the same category were selected, which were commonly used and with an ordinary design. Finally, 35 subjects evaluated these 12 products using a perceived uniqueness scale [11]. Each unique product was perceived as higher on uniqueness than their counterparts (ps < .001). For the pairs containing university-related products we observed no such differences (ps > .05).

**Development of the social identity threat manipulation**

To induce a threat to social identity, we adopted the previously successfully used method of providing negative group feedback [9,10,12]. University social identity was threatened, which is an essential and salient aspect of students' social identity [13]. Social identity was threatened by providing information about the low level of professional competence of students of the university from which the subjects came in comparison to students of other universities [9,12]. The pretext justifying such information was that the subjects performed the Test of Professional Competence. This test aimed to increase the importance of professional competencies for the subjects, and the ambiguous structure of the answers was to highlight its difficulty. Participants answers to fifteen questions from multiple-choice "Oxford Test of Professional Competence" (OTPC), described as a "test which allows with great accuracy to predict the future level of functioning on the labor market." They then found out that the test was used in the Perspective - Graduates On The Work Market study conducted on a group of 19,000 students in Poland whose professional careers had been monitored for five years after graduation. After completing the test, the respondents entered the university's name in which they were studying to learn the results of students in this test. Subsequently, the respondents were informed that out of 40 possible credits, students from their universities scored on average 13.1 (low scores), and the overall average for students was 27.3. Finally, they received the description of low scores in the OTPC: “LOW RESULT – description: The attitudes towards work of the group of people who received scores in the low score range indicate potential difficulties in the future in finding and keeping a satisfying job. Students who scored low on this test during their studies were often not valued as employees and found it difficult to complete assigned tasks in a reliable and timely manner. Students who scored low on the test had difficulty in learning from their mistakes and did not pay enough attention to the feedback they received from their superiors. They were characterized by poor consistency and persistence in their work. Low scores are mainly given to people with insufficient motivation to work and low commitment to their work.”

Participants in the control condition read a neutral description of the test: “The test measures attitudes toward work and predicts career preferences. The test allows determining such preferences as: a preferred way of fulfilling assigned tasks; a preferred way of drawing conclusions from one's mistakes; attitude towards feedback received from superiors; type of consistency and persistence in performing work; type of motivation to work, and involvement in the work performed.”

We conducted a pretest of social identity threat manipulation, in which we threatened women's social identity (not university social identity, as we did in the main study), using an analogous procedure. 42 undergraduates females were randomly assigned into two conditions: feminine social identity threat (n = 22) vs. no threat (n = 20). We excluded two individuals from the analysis because they unmasked the purpose of the study. To assess the relative importance of professional competence to the social identity, we asked participants to rate before manipulation of identity threat how important this competence was them as to women (social identity). Participants rated the importance of professional competence on a 7-point scale ranging from 1 (not at all important) to 7 (very important). Participants, on average, "rather agree" with the statement that professional competence is an important part of their social identity (M = 4.74, SD = 1.09).

To assess the effectiveness of manipulation of the social identity threat, we asked participants to indicate the extent to which they felt threatened by eight adjectives (α = .91): "threatened," "concerned," "calm" (reversed item), "nervous," "upset," "frightened," "jittery" and "uncertain." We also used the private subscale Collective Self-Esteem scale at state levels (α = .90) [14]. A lower level of private collective self-esteem is an indicator of a social identity threat. [15] To assess whether the feeling of anxiety was aroused, the State-Trait Anxiety Inventory [16,17] was used, a 20-item self-reported questionnaire that measures state anxiety (STAI-S) at the moment of scoring with four-point Likert scales (α = .81).

Results revealed that significantly greater threat was experienced in the social identity threat condition (M = 2.24, SD = 1.02) as opposed to the no-threat (M = 1.60, SD = 0.51) condition, t(38) = -2.51, p < .05, d = .79. In the social identity threat condition was observed lower level of private collective self-esteem (M = 4.53, SD = 1.66) as compared to the no-threat (M = 5.75, SD = 1.03) condition, t(38) = -2.81, p < .01, d = .88. Results demonstrated that significantly greater anxiety was experienced in the social identity threat condition (M = 1.82, SD = 0.42) as opposed to the no-threat (M = 1.56, SD = 0.32) condition, t(38) = -2.21, p < .05, d = .70. These results suggest that our manipulation of social identity threat and induction of anxiety were effective.

**References**

1. Kühnen U, Hannover B. Assimilation and contrast in social comparisons as a consequence of self-construal activation. EUR J SOC PSYCHOL. 2000;30(6): 799-811.
2. Oishi S, Wyer jr RS, Colcombe SJ. PERS SOC PSYCHOL. 2000;78(3): 434–445.
3. Stapel DA, Koomen W. I, we, and the effects of others on me: how self-construal level moderates social comparison effects. J PERS SOC PSYCHOL. 2001;80(5): 766–781.
4. Singelis, T.M. The measurement of independent and interdependent self-construals. PERS SOC PSYCHOL B. 1994;20(5): 580-591.
5. Kuhn MH, McPartland TS. An empirical investigation of self-attitudes. AM SOCIOL REV. 1954;19(1): 68-76.
6. Knowles ML, Gardner WL. Benefits of membership: The activation and amplification of group identities in response to social rejection. PERS SOC PSYCHOL B. 2008;34(9): 1200-1213.
7. Brewer MB, Gardner WL. Who is this" We"? Levels of collective identity and self representations. J PERS SOC PSYCHOL. 1996;71(1): 83-93.
8. Tian KT, Bearden WO, Hunter GL. Consumers' need for uniqueness: Scale development and validation. J CONSUM RES. 2001;28(1): 50-66.
9. White K, Argo JJ. Social identity threat and consumer preferences. J CONSUM PSYCHOL. 2009;19(3): 313-325.
10. White K, Argo JJ, Sengupta J. Dissociative versus associative responses to social identity threat: The role of consumer self-construal. J CONSUM RES. 2012;39(4): 704-719.
11. Song D, Lee J. Balancing "We" and "I": Self‐construal and an alternative approach to seeking uniqueness. J CONSUM BEHAV. 2013;12(6): 506-516.
12. Brockner J, Chen YR. The moderating roles of self-esteem and self-construal in reaction to a threat to the self: Evidence from the People's Republic of China and the United States. J PERS SOC PSYCHOL. 1996;71(3): 603–615.
13. LeBoeuf RA, Shafir E, Bayuk JB. The conflicting choices of alternating selves. ORGAN BEHAV HUM DEC. 2010;111(1): 48-61.
14. Luhtanen, R, Crocker, J. A collective self-esteem scale: Self-evaluation of one's social identity. PERS SOC PSYCHOL B. 1992;18(3): 302-318.
15. Scheepers D, Ellemers N. When the pressure is up: The assessment of social identity threat in low and high status groups. J EXP SOC PSYCHOL. 2005;41(2): 192-200.
16. Spielberger CD, Gorsuch RL, Lushene PR, Vagg PR, Jacobs AG. Manual for the State-Trait Anxiety Inventory (Form Y). Palo Alto: Consulting Psychologists Press; 1983.
17. Wrześniewski K, Sosnowski T, Jaworowska A, Fecenec D. Polish adaptation of STAI Manual, 3rd extended edition. Warszawa: Pracownia Testów Psychologicznych PTP; 2006.
